# Editorial: The psychological challenges of remote working

**DOI:** 10.3389/fpsyg.2023.1190064

**Published:** 2023-05-31

**Authors:** Naval Garg, Freda Van der Walt, John Burgess

**Affiliations:** ^1^School of Management and Entrepreneurship, Delhi Technological University, Rohini, India; ^2^Department of Business Management, Central University of Technology, Bloemfontein, Free State, South Africa; ^3^Centre for Organisational Change and Agility, Torrens University Australia, Adelaide, SA, Australia

**Keywords:** remote work, COVID, psychology, challenges, mental health

Prior to the emergence of the gig economy there were industries that involved remote working and extended commuting such as mining and construction; and mobile work driven by telephony, long distance commuting and internet access. Such arrangements contributed to exhaustion, loneliness and family breakdown (Berg et al., 2018). In the gig economy, platform mediated work, whether in its physical form such as food delivery services or through online data analysis has transformed work and industries globally (Ayentimi et al., 2023). Conditions supporting remote and online working include the increasing access to the internet, smart phones and their related technologies, software and cloud computing developments, the rise of global online service providers and the ease of conducting business and shopping online (Wheatley et al., 2021). The significant technological transformation associated with the fourth industrial revolution technologies (Schwab, 2015) has supported working from home, remote working, and mobile work from locations that have internet access.

Interest in examining the psychological effects of remote working was driven by the COVID-19 pandemic that forced millions into work-from-home work arrangements (Kniffin et al., 2021). It represented a significant change in lifestyle, working and living habits. This monumental shift was unplanned and implemented without knowledge of the potential challenges associated with prolonged periods of homeworking (Kowal et al., 2020). This represented one of the most significant changes in working arrangements globally and provided the opportunities for researchers, organization, and governments to assess the consequences of extended working from home arrangements. Researchers have examined the crucial issues that include the impact on employee stress and wellbeing (Saladino et al., 2020; Spagnoli et al., 2020); the management of the workforce and the impact on managers (Carnevale and Hatak, 2020; Ipsen et al., 2022); family and household challenges including work-family conflict (Prikhidko et al., 2020); and employee motivation and engagement (Galanti et al., 2021).

Following COVID, remote and homeworking arrangements will remain embedded in organizational employment practice and it is important to assess he psychological effects of extended remote work for workers, managers, organizations, and households/families. Potential personal challenges include isolation, the blurring of work-life boundaries, surveillance, and being on call (Wheatley et al., 2021). In turn, programs and policies that reduce the psychological risks are essential for organizations and policymakers as remote and home working is extended across all industries. This Research Topic provides a timely, diverse, and detailed examination of the psychological impacts of remote work. Within this Research Topic, nine articles examine the above issues and experiences across countries through surveys of workers, households, and managers. Each article provides thoughtful suggestions for policies to support remote working and reduce its psychological risks.

Bodini et al. reported positive work-from home benefits across life and work domains among Italian knowledge workers following 18 months of working from home. Explanatory factors that contributed to these positive impacts included home to work commute times, changes in lifestyle arrangements and work-room sharing. The results indicate that inclusion and a sense of community are in improving workers' health and in offsetting the impact of isolation from working at home.

Carvalho et al. assess the impact of boundary controls on the relationship between family-supportive supervision behaviors (FSSB) and life satisfaction for teleworking. They also examine the moderating effect of the country on the relationship (Pakistan vs. Portugal). FSSB was e important to control teleworker boundaries and was linked to life satisfaction FFSB was found to contribute to higher levels of life satisfaction.

He et al. examined the impact of home working on families in China. They indicate that proactive/passive work connectivity behaviors support family harmony through self-efficacy and ego depletion. They r explore the moderating role of family support on this relationship.

Lescarret et al. investigated the determinants of employees' intention to telework in a coworking space, From an online survey of French teleworkers, they found that the perceived lack of working comfort while teleworking impacted the perceived usefulness of teleworking in a coworking space and also affected their future intentions to telework in a coworking space.

Maden-Eyiusta and Alparslan demonstrated a mediating role for psychological empowerment in the relationship between self-leadership and work role performance in remote work settings in Turkey. They also demonstrated partial support for the moderating role of supervisor close monitoring of employees. The study outlines the motivational process through which self-leadership results in improve work role performance.

Potgieter and Ferreira, through a survey of African and European participants, reported a close association between career adaptability and career wellbeing and the perceived value-orientated psychological contract.

Redaelli et al. investigated to what extent perceived COVID-19-crisis intensity (PCCI) results in parental burnout as manifested through exhaustion, emotional detachment from one's children and sense of parental inefficacy. The mediating role of work–family conflict (WFC) and the buffering effects of family-supportive organizational perceptions (FSOP) during the pandemic were also explored.

Tautz et al. investigated transformational leadership and health-oriented leadership among two cohorts (leaders and employees) in remote work settings in Germany. Both groups were asked to report their experiences of working from home as compared to working in traditional office settings. Participants reported that lack of social presence, limited informal chats, communication difficulties and a lack of mutual trust that inhibits transformational and health-oriented leadership.

van Gelder et al., reported that workplace innovation in the context of the Netherlands is positively associated with engagement via its effect on meaningful work but that it is not associated with exhaustion. Work–life segmentation preference amplifies the relationship between meaningful work and engagement and exhaustion. Line managers with strong work–life segmentation preference and a low score on meaningful work experience have less engagement and more exhaustion than line managers with a high score on meaningful work when working from home.

## Author contributions

All authors listed have made a substantial, direct, and intellectual contribution to the work and approved it for publication.
